# Evaluation of remodeling and geometry on the biomechanical properties of nacreous bivalve shells

**DOI:** 10.1038/s41598-021-04414-1

**Published:** 2022-01-13

**Authors:** Estefano Muñoz-Moya, Claudio M. García-Herrera, Nelson A. Lagos, Aldo F. Abarca-Ortega, Antonio G. Checa, Elizabeth M. Harper

**Affiliations:** 1 Departamento de Ingeniería Mecánica, Universidad de Santiago de Chile (USACH), Av. Bernardo O’Higgins 3363, Santiago de Chile, Chile; 2 Centro de Investigación e Innovación para el Cambio Climático (CiiCC), Universidad Santo Tomás, Av. Ejército Libertador 146, Santiago de Chile, Chile; 3 Center for Biomedical Technology, Universidad Politécnica de Madrid, 28223 Pozuelo de Alarcón, Spain; 4 Departamento de Estratigrafía y Paleontología, Universidad de Granada, 18071 Granada, Spain; 5 Department of Earth Sciences, University of Cambridge, Downing Street, Cambridge, CB2 3EQ UK

**Keywords:** Bioinspired materials, Mechanical engineering, Biophysics, Materials science

## Abstract

Mollusks have developed a broad diversity of shelled structures to protect against challenges imposed by biological interactions(e.g., predation) and constraints (e.g., $pCO_2$-induced ocean acidification and wave-forces). Although the study of shell biomechanical properties with nacreous microstructure has provided understanding about the role of shell integrity and functionality on mollusk performance and survival, there are no studies, to our knowledge, that delve into the variability of these properties during the mollusk ontogeny, between both shells of bivalves or across the shell length. In this study, using as a model the intertidal mussel *Perumytilus purpuratus* to obtain, for the first time, the mechanical properties of its shells with nacreous microstructure; we perform uniaxial compression tests oriented in three orthogonal axes corresponding to the orthotropic directions of the shell material behavior (thickness, longitudinal, and transversal). Thus, we evaluated whether the shell material’s stress and strain strength and elastic modulus showed differences in mechanical behavior in mussels of different sizes, between valves, and across the shell length. Our results showed that the biomechanical properties of the material building the *P. purpuratus* shells are symmetrical in both valves and homogeneous across the shell length. However, uniaxial compression tests performed across the shell thickness showed that biomechanical performance depends on the shell size (aging); and that mechanical properties such as the elastic modulus, maximum stress, and strain become degraded during ontogeny. SEM observations evidenced that compression induced a tortuous fracture with a delamination effect on the aragonite mineralogical structure of the shell. Findings suggest that *P. purpuratus* may become vulnerable to durophagous predators and wave forces in older stages, with implications in mussel beds ecology and biodiversity of intertidal habitats.

## Introduction

The mollusk shell is a biomaterial composed of organic and mineral phases, respectively, in a 1–5$\%$ and 99–95$\%$^1,2^. The mineral phase in carbonate takes the form of calcite or aragonite, both crystalline polymorphs of calcium carbonate^2^. In the case of bivalves, the shell microstructure may have various configurations or combinations^3^. One of the most ubiquitous is nacre with a laminar brick-and-mortar-like structure^1^, in which pseudohexagonal tablets arrange into lamellae. The different lamellae are separated by soft organic membranes, called interlamellar membranes^4–6^. Nacre is broadly present in mollusks such as gastropods, bivalves, cephalopods, and monoplacophorans. It has a complex hierarchical architecture encompassing multiple length scales from the nanoscale to the macro-scale^4,7–10^. Thus, the organic and the mineral phase provide different structural and functional properties to the mollusk shell^11^. Such is the advantage of nacre to mechanical stimuli that the brick-and-mortar architecture of biological nacre has inspired the development of synthetic composites with enhanced fracture toughness and multiple functionalities^1,12–14^.

The study of mechanical properties and strength of bivalve shells has been conducted to observe adverse effects produced by the environment, such as climate change or predators. For instance, the effects of ocean acidification and warming affected the shell strength^15^ and fracture resistance of *Mytilus edulis*^16^. In addition, studies indicate that the predator gastropod *Concholepas concholepas* attacks the valves of the mussel *P. purpuratus* using a drilling behavior^17^ and that the risk exposure to shell crushing predators affects the thickness of mussels shells^18^. However, even though the study of shell biomechanical properties has provided understanding about the role of shell integrity and functionality on mollusk performance and survival, few studies have considered a detailed analysis of the variability of these properties during the mollusk ontogeny, between both shells of bivalves or across the overall shell. In general, biomechanical studies implicitly assume that mechanical properties are stable through the factors mentioned above for nacre microstructures. For instance, the mechanical behavior of pearls and shells with spherical and flat laminations of the bivalve *Sinanodonta woodiana* was studied and compared with that of geological aragonite, although without specifying which section or valve was used in the analysis^19^. Other studies performed structure and mechanical behavior of the bivalve *Saxidomus purpuratus* shell using compression tests and comparing between different planes of the shell material, without any specification about the valves used for the experiment^20^. Meyers et al. carried out tensile and compression tests in nacreous material structures without specifying which valve was used^6^. Ji et al. observed differences in mechanical properties in different shell sectors of *Clinocardium californiense*^21^. However, the microstructure analyzed by the authors corresponds to a crossed-lamellar and not to a nacreous microstructure, as studied in this research.

Concerning ontogeny, mollusks’ shells provide support and protection throughout the animal’s life. During the growth process of the mussel, several changes occur in the mechanical properties of the material, an effect called “remodeling”. These changes, often adaptive, may be brought about by alterations in stiffness, internal structure, strength, or density^22^. Studies have focused on the mechanics of the nacreous material of bivalves, in which tensile, compression, shear, indentation, bending, torsion, and scratching tests have been performed^1,23–25^, including fracture analysis of the material^26–32^. Mohanty et al. reported the nanomechanical behavior of nacre (using the gastropod *Haliotis rufescens* shell), showing that aragonite platelets have a viscoelastic behavior when organic material is present due to their wet state^33^, and such behavior may well change as that organic material either decays or denatures with age. Bezares et al. indicated that the hardness was much lower under the presence of moisture than in the dry state, producing plastic deformation at the edges, displaying anisotropic behavior^34^. Thus, in a broad biomechanical perspective, whether shell valves have similar mechanical behavior throughout ontogeny or whether their valves are symmetrical and homogeneous remains open. Therefore, to fill these knowledge gaps, we contribute to understanding the biomechanical behavior of nacre, selecting the mussel *P. purpuratus* as a species model.

The bivalve *Perumytilus purpuratus* has a nacreous sheet structure^35,36^ and is the dominant competitor of the rocky substrate^37,38^ in wave-exposed rocky intertidal habitats along the Peruvian and Chilean coasts^39^. The shells of this mussel are aggregated, building dense three-dimensional matrices that provide biogenic habitat to up to 92 intertidal species, which find refuge from wave action and predators within the matrices^40^. Thus, *P. purpuratus* has the role of ecosystem engineer species in the intertidal zones, increasing the local biodiversity^41^. Due to this relevant ecological role of *P. purpuratus*, many studies have been carried out on the effects of predators^17,42–47^ and environmental factors, such as climate change^48–54^. However, these studies have assumed homogeneity of mechanical properties and symmetry of both shells, so the effects of predators may not be entirely clear. In addition, the mechanical properties of the shell of *P. purpuratus* have not yet been characterized.

This study aims to characterize, for the first time, the anisotropic biomechanical behavior of *P. purpuratus* shells at the macro level. Our goal is to evaluate whether there are differences in the material’s mechanical properties between the individual’s different shell sizes (aging), between the left and right valve, and across the shell length. For this purpose, uniaxial compression tests have been performed in three directions of the material corresponding to orthotropic behavior, considering an anisotropic linear elastic model. The failure characteristics of the compressed samples were observed with a scanning electron microscope (SEM). We discuss the results about the mechanical properties of mollusk shells and the methodological implications of variation in biomechanical properties of the valves, section, and mussels shell sizes.

## Materials and methods

### Sample collection

**Figure 1:**
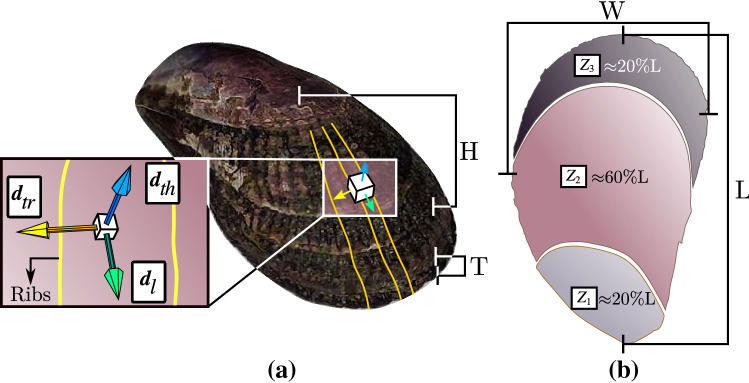
*L*: length, *H*: height, *W*: width, *T*: thickness. (**a**) Orthotropic directions of *P. purpuratus* shell material behavior: thickness direction ($\pmb {d}_{th}$), longitudinal direction ($\pmb {d}_{l}$) and transversal direction ($\pmb {d}_{tr}$), (**b**) Zones in which the shell was divided, Zone 1 ($Z_1$), Zone 2 ($Z_2$) and Zone 3 ($Z_3$). The respective percentage of the length is presented for each zone.

Individuals of *P. purpuratus* were randomly collected in July 2019 from the intertidal habitats of the coastal area of Huasco (28° 30′ S; 71° 15′ W; Chile). In the laboratory, individuals were characterized by their weight and linear dimensions (Table 1). After removing the soft tissues, the shells were dried at room temperature to perform the tests with the shells in a dry state. Then, left and right valves were processed separately. The length, width, height, and thickness of the right shell (Fig. 1), as defined by Manríquez et al.^17^, were measured with a digital Vernier Calliper (Mitutoyo ± 0.01 mm). The weights of the left and right valves were measured in an analytical balance (SHIMADZU AUX120 ± 0.0001 g), as shown in Table 1. Mussels were processed in compliance with the principles of laboratory animal care. The Ethics Committees approved all animal care and experimental procedures of the Universidad Santo Tomás and the Universidad de Santiago de Chile (IE N$^\circ$ 0146). They were conducted according to the Guide for the Care and Use of Laboratory Animals published by the US National Institutes of Health (NIH Publication No. 85-23, revised 1996).

**Table 1:** Mean value and the standard error of the mean (±SEM): length, height, width, the thickness of the right shell, left valve weight, right valve weight.

Group	Length (mm)	Height (mm)	Width (mm)	Thickness (mm)	Left valve weight (g)	Right valve weight (g)
1	14.42 ± 0.30	6.55 ± 0.14	7.67 ± 0.40	0.40 ± 0.03	0.15 ± 0.01	0.15 ± 0.01
2	19.92 ± 0.44	10.55 ± 0.65	10.70 ± 0.44	0.47 ± 0.05	0.37 ± 0.03	0.38 ± 0.02
3	26.18 ± 0.64	11.77 ± 0.60	14.15 ± 0.80	0.59 ± 0.06	0.83 ± 0.11	0.82 ± 0.11
4	32.44 ± 1.30	15.13 ± 0.51	14.96 ± 0.23	0.79 ± 0.03	1.57 ± 0.15	1.60 ± 0.21
5	37.55 ± 1.61	18.46 ± 0.60	16.76 ± 0.32	0.72 ± 0.03	2.71 ± 0.02	2.77 ± 0.08

**Figure 2:**
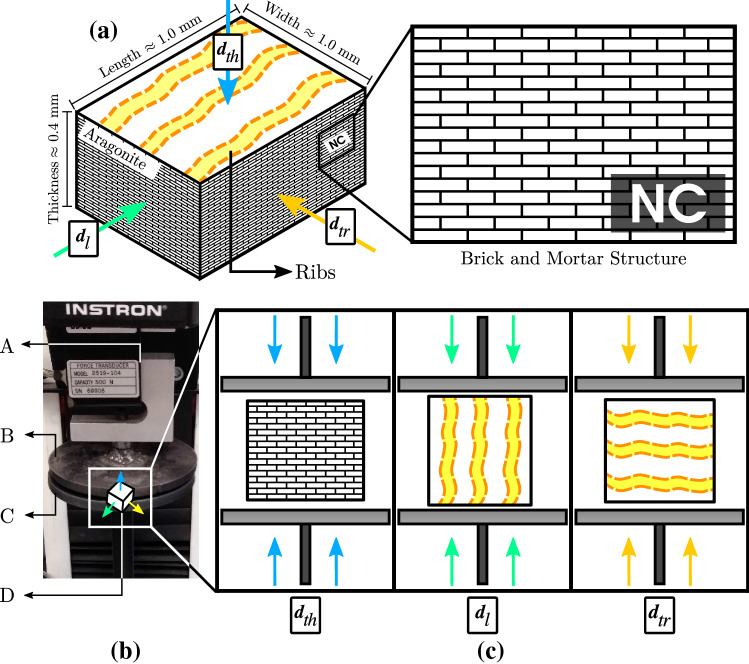
Uniaxial compression test assembly. (**a**) Orthotropic directions of the nacreous material (brick-and-mortar structure) represented in the sample used in uniaxial compression test. NC: nacreous layer cross-section, (**b**) A: load cell, B: mobile top plate, C: fixed bottom plate, D: sample, (**c**) arrangement of the samples according to the direction of compression.

The mussels were divided into five groups (three individuals per group) based on shell length, representing nominal growth stages from juvenile until maturity^37,53,55,56^. To observe differences in mechanical properties as the shell grows, the valves were divided into three zones ($Z_1$, $Z_2$, and $Z_3$) along the shell length (Fig. 1b). $Z_3$ is located at the shell margin (posterior), $Z_1$ corresponds to the sector closest to the umbo (anterior), and $Z_2$ is the sector between $Z_1$ and $Z_3$. From each valve and shell section, three samples (Fig. 2a) were extracted to be compressed in the three directions of the material’s mechanical behavior (Fig. 1a): thickness direction ($\pmb {d}_{th}$), which is normal to the surface of the shell; longitudinal direction ($\pmb {d}_{l}$), according to the growth direction, and parallel to the ribs of the shell; transversal direction ($\pmb {d}_{tr}$), perpendicular to the shell ribs. These directions have been previously evaluated in other bivalves under compression tests^1,20^.

### Experimental procedure

Uniaxial compression tests were performed on an Instron 3342 universal testing machine. This test compresses a prism-shaped sample of the shell material with an exposed nacreous layer cross-section (NC), whose orientations are parallel to the orthotropic directions of the material. The samples were obtained from each shell zone with a Dremel 3000 2/30 ACC multi-purpose cutting tool. The shell cuts were made in sectors far away from where the samples were extracted so that the pieces were not directly affected by the cutting tool. Then, the faces of the sample were sanded to obtain the dimensions shown in Fig. 2a, measured using a digital Vernier Calliper described above. The parallelism of the sample faces was checked with a High Accuracy Digital Micrometer CNC sensor (Keyence LS-7070 MT ± 3 $\mu$m). Before performing the compression test, each sample was observed through a stereomicroscope (SMZ-161-BLED; magnification range: 7.5x-45x) to observe possible surface fractures in the samples, and thus, discard it and extract a new one.

The tests were performed using dry shells and applying a compression load (Fig. 2b), with a constant displacement speed of 0.05 [mm/min], in the three orientations described above (Fig. 2c). In addition, the reaction force from the mobile plate’s imposed displacement was recorded by the load cell (Instron: 2519 series static load cells 500 N) (Fig. 2b). Thus, a total of 270 compression tests were performed (90 per direction).

After the test, load cell force and displacement data (*f* and *d*) were acquired. The stress-strain curve ($\sigma$-$\varepsilon$) was calculated as follows: $\sigma = F/A_0$ and $\varepsilon = \Delta l/l_0$, where $A_0$ is the initial cross-sectional area of the sample, normal to the loading direction ($\pmb {d}_{th}$, $\pmb {d}_{l}$, and $\pmb {d}_{tr}$), $l_0$ is the initial length of the sample, and $\Delta l$ is the length variation of the sample during the test, according to the geometry of the sample (Fig. 2a). The data allowed us to know its mechanical properties considering a linear orthotropic behavior: elastic modulus (*E*), maximum stress before the failure ($\sigma _{max}$), and maximum strain before the failure ($\varepsilon _{max}$).

### Scanning electron microscopy (SEM)

Scanning Electron Microscopy (SEM) was performed on the shell samples used in the uniaxial compression tests, specifically those compressed in the longitudinal and transversal directions (see Fig. 1). A gold coating of approximately 30 nm in thickness was applied using the Cressington 108 Auto Sputter Coater equipment. Gold coating application is a common rule, and its main bands are located at 2.1 KeV and 9.7 KeV. The examination was performed in a Zeiss EVO MA 10 at 20 kV.

### Statistical methods

To evaluate differences, we used an Analysis of Covariance (ANCOVA), using the shell thickness of each individual as a covariate. These mechanical properties were compared between the valves (left and right), shell age/size (groups 1 to 5, Table 1), and three shell zones ($Z_1$, $Z_2$, and $Z_3$) of each valve (Fig. 1b), considering these factors as fixed. Tukey’s test was performed to identify which levels of main factors generated the significant differences. ANCOVA assumptions were evaluated using the residues of the entire model. The analysis was implemented using GLM (general linear model). Both the residual graph and the variance heterogeneity test showed no significant deviations.

## Results and discussion

### Uniaxial compression test

**Figure 3:**
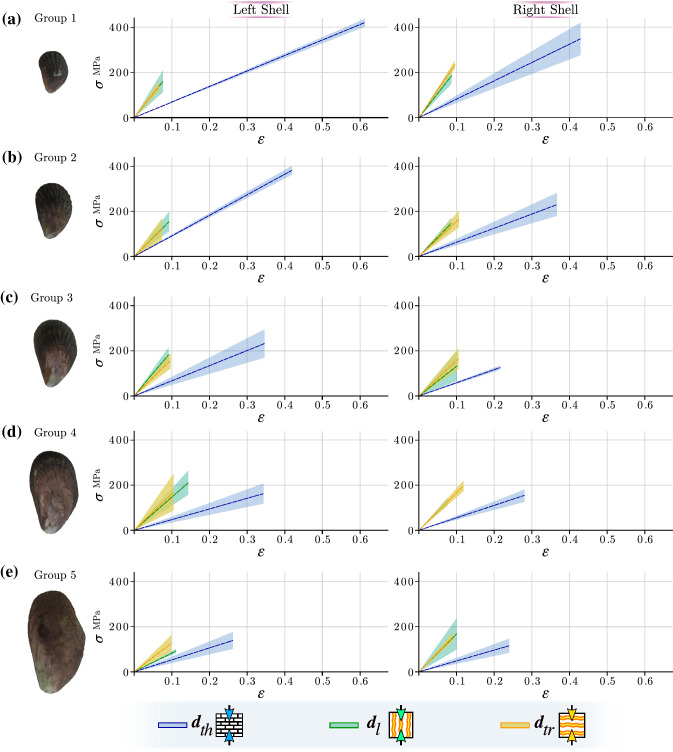
Standard deviation (SD) of the stress–strain curve ($\sigma -\varepsilon$) of the five age/size groups (along with a photograph of the right valve representative of each group) for the left and right shells in their respective orthotropic directions of the material: thickness ($\pmb {d}_{th}$), longitudinal ($\pmb {d}_{l}$) and transversal ($\pmb {d}_{tr}$) direction. (**a**) group 1, (**b**) group 2, (**c**) group 3, (**d**) group 4, (**e**) group 5.

Regression analysis of the compression tests evidenced a linear elastic behavior of the *P. purpuratus* shells (Fig. 3). It is also observed that the shell material is brittle, i.e., without appreciable plastic deformation, so the curve only represents the elastic zone in the stress-strain diagram.

**Figure 4:**
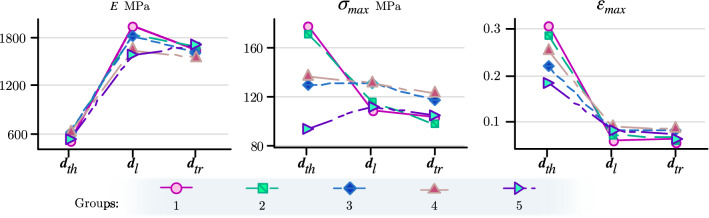
Average values of elastic modulus (*E*), maximum stress ($\sigma _{max}$), and maximum strain ($\varepsilon _{max}$) for five groups of sizes/ages in the three orthotropy directions: thickness ($\pmb {d}_{th}$), longitudinal ($\pmb {d}_{l}$), and transversal ($\pmb {d}_{tr}$).

The regression lines show an apparent symmetry of the mechanical properties between the left and right valves for all age/size groups and a decrease or deterioration in maximum stress and maximum strain across the thickness plane as the mollusk increases in size. The compression tests performed in the other two directions do not show an observable deterioration in their mechanical properties, as shown in Fig. 4, which shows the average values of the mechanical properties for each size. Detailed information of the mechanical properties (values and standard deviation) can be found in Supplementary Table S1.

#### Statistical results

Elastic modulus, maximum stress, and maximum strain showed no significant differences when comparing shell valves and the three shell zones of *P. purpuratus* (ANCOVA, Table 2). However, we found significant differences between shell size and shell thickness in elastic modulus and maximum strain and significant differences in the three mechanical properties evaluated across the three directions of the compression test. Therefore, there was a significant difference between at least two directions of orthotropy of the shell material, but these differences do not interact with variations of the mechanical properties of the shell zones. The direction interacts significantly with the size of the shell and valves (Table 2). Boxplots of the mechanical properties for both valves can be found in Supplementary Fig. S1.

**Table 2:** Results of ANCOVA analysis for elastic modulus (*E*), maximum stress ($\sigma _{max}$), and maximum strain ($\varepsilon _{max}$).

Sources of variation	Elastic modulus (*E*)			Maximum stress ($\sigma _{max}$)			Maximum strain ($\varepsilon _{max}$)		
	*Df*	*f*	*p*	*Df*	*f*	*p*	*Df*	*f*	*p*
Thickness (*T*)	1, 179	36.85	**< 0.001**	1, 179	21.82	**< 0.001**	1,179	1.69	0.195
Size (*S*)	4, 179	9.69	**< 0.001**	4, 179	6.62	**< 0.001**	4, 179	2.03	0.093
Left-right valve (*V*)	1, 179	1.71	0.193	1, 179	2.12	0.147	1, 179	2.62	0.107
Zone (*Z*)	2, 179	1.87	0.158	2, 179	0.2	0.819	2, 179	1.01	0.36
Direction (*d*)	2, 179	170.27	**< 0.001**	2, 179	4.81	**0.009**	2, 179	153.96	**0.001**
$S \times V$	4, 179	0.79	0.531	4, 179	0.46	0.763	4, 179	0.52	0.721
$S \times Z$	8, 179	0.47	0.873	8, 179	1.63	0.120	8, 179	1.54	0.145
$S \times d$	8, 179	0.8 ara>	0.605	8, 179	1.68	0.106	8, 179	3.33	**0.001**
$V \times Z$	2, 179	0.52	0.598	2, 179	0.39	0.678	2, 179	0.65	0.526
$V \times d$	2, 179	0.31	0.733	2, 179	2.06	0.131	2, 179	3.14	**0.045**
$Z \times d$	4, 179	1.53	0.197	4, 179	1.84	0.124	4, 179	0.89	0.469
$S \times V \times Z$	8, 179	0.1	0.999	8, 179	0.46	0.883	8, 179	0.5	0.854
$S \times V \times d$	8, 179	0.4	0.920	8, 179	0.82	0.583	8, 179	1.49	0.165
$S \times Z \times d$	16, 179	0.89	0.578	16, 179	0.9	0.575	16, 179	1.03	0.429
$V \times Z \times d$	4, 179	1.07	0.374	4, 179	1.02	0.399	4, 179	0.28	0.891
$S \times V \times Z \times d$	16, 179	0.32	0.995	16, 179	0.86	0.620	16, 179	0.57	0.903

The shell thickness (*T*) mm of each individual was used as a covariate. Five size ranges (*S*), the left and right valves (*V*), the three shell zones (*Z*), and three compression directions (*d*) were considered.

*Df*, degrees of freedom; *f*, f-statistic; *p*, *p-value*.

Significant p values ($<0.05$) are shown in bold.

Tukey’s tests were performed a *posteriori* and revealed no significant differences between the mechanical properties (*E*, $\sigma _{max}$, and $\varepsilon _{max}$) of the longitudinal and transversal directions ($p>0.05$). However, there were significant differences between thickness direction, with the longitudinal and transversal directions ($p<0.05$). *P. purpuratus* sizes revealed significant differences in mechanical properties in the three orthotropic directions ($p<0.05$), i.e., a decrease in the magnitude of the mechanical properties until size 5 (Fig. 3). Therefore, in general, the three mechanical properties for each direction present a greater magnitude in the first three sizes, to decrease and be constant in sizes 4 and 5 subsequently. Also, it is observed that the thickness direction showed increased elasticity in the juvenile mussel sizes, both in the maximum stress and maximum strain. Nevertheless, the maximum stress decreases for large sizes, and its values reach lower magnitudes than the other directions at the same age. Therefore, the material becomes more brittle as the mussels increase in shell size (age), specifically across the thickness direction.

Mechanical properties of compression performed across the thickness direction vary among groups due to the remodeling process from the youngest to the oldest individuals (groups 1 to 5), as follows: $\sigma _{max}$ = 178 - 93 MPa and $\varepsilon _{max} = 0.3 - 0.18$. However, the elastic modulus does not present such notable variations; the results show that the magnitudes range from 495 to 596 MPa. The other directions ($\pmb {d}_{l}$ and $\pmb {d}_{tr}$) show no difference in the mechanical properties between them and do not vary greatly as mussels increase in shell size. The average mechanical properties for both directions are as follows: $\sigma _{max} = 126$ MPa and $\varepsilon _{max}$ = 0.13; the elastic modulus varies between the different ages (groups 1–5): *E* = 1790–1655 MPa. The average values of the mechanical properties of each size and direction of the shells can be seen in Supplementary Table S1.

Several studies are related to the mechanical properties of mollusk shells obtained in quasi-static compression tests for shell materials with nacreous microstructure in a dry state, specifically in the gastropod class, such as *Haliotis rufescens* (abalone), studied in the work of Menig et al.^57^, and compiled (together with other works on nacre) in the review made by Sun and Bhushan^1^. The values reported correspond to the mechanical properties in the thickness, longitudinal, and transversal directions (the latter two without distinction). For the thickness direction, the maximum stress of abalone ranged from 250 to 540 MPa. On the other hand, the longitudinal and transversal directions presented maximum stress values of approximately 235–548 MPa. Therefore, the mechanical properties of *P. purpuratus* differ from those of *Haliotis rufescens*. The thickness direction of *P. purpuratus* presented lower maximum stress than that supported by the abalone. In contrast, the maximum stress supported by the shell material of abalone in the longitudinal and transversal directions is notably lower, approximately 13$\%$ of that supported by *P. purpuratus*.

#### SEM observations

**Figure 5:**
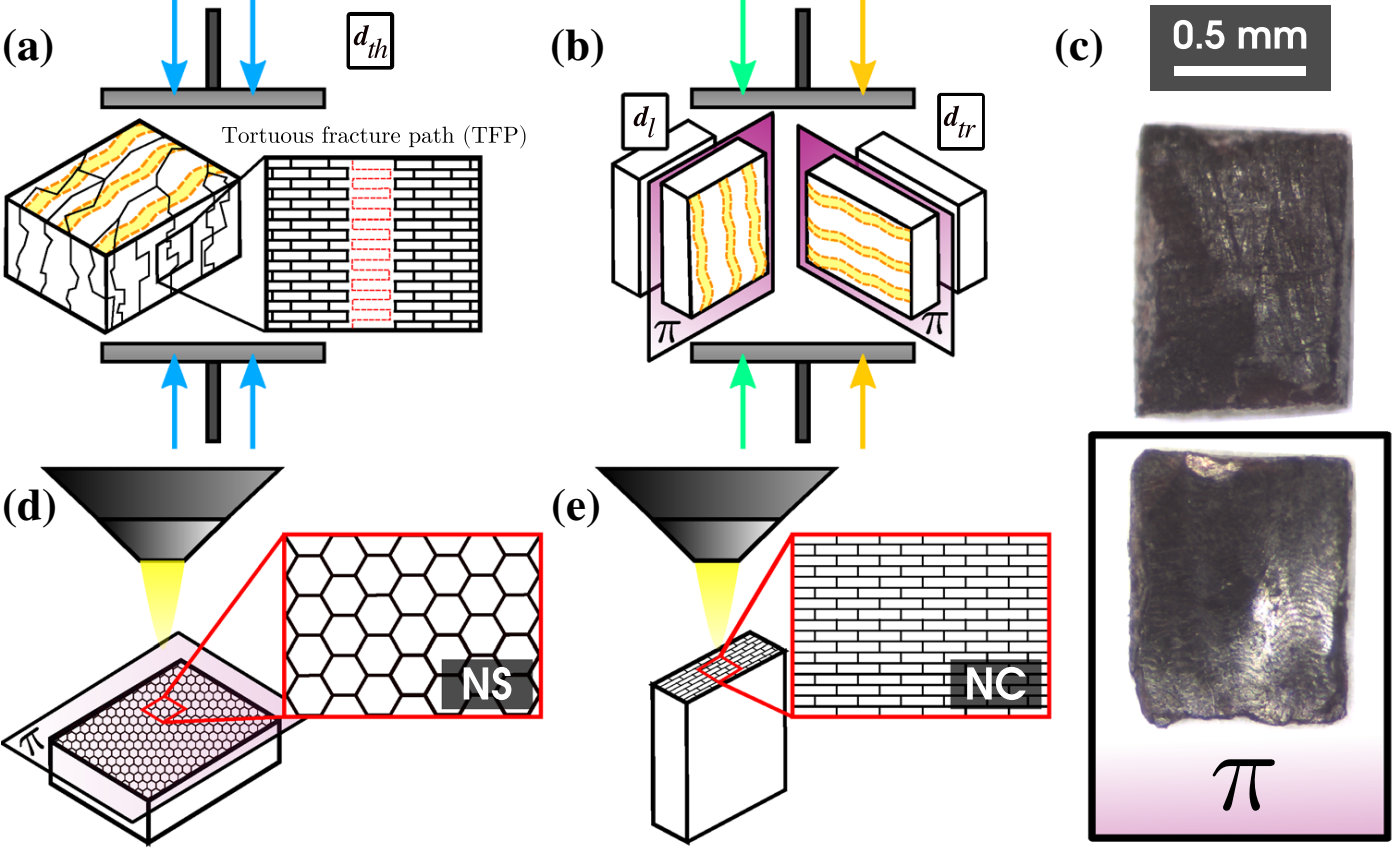
Failure characteristic of the sample at the end of the uniaxial compression test in the thickness ($\pmb {d}_{th}$), longitudinal ($\pmb {d}_{l}$), and transversal ($\pmb {d}_{tr}$) directions. (**a**) fractured sample by failure with an apparently tortuous fracture at the end of the test in the thickness direction ($\pmb {d}_{th}$), (**b**) fractured sample by delamination at the end of the compression test in the longitudinal and transversal directions ($\pmb {d}_{l}$ and $\pmb {d}_{tr}$), (**c**) Photograph of fractured sample after the compression test in the longitudinal and transversal directions ($\pmb {d}_{l}$ and $\pmb {d}_{tr}$), taken with a stereomicroscope (SMZ-161-BLED; magnification range: $7.5\times-45\times$) and a microscope camera (Motic Moticam 2.0 MP), (**d**) Arrangement of samples for nacreous layer surface (NS), (**e**) Arrangement of samples for nacreous layer cross-section (NC). $\pi$: delamination plane.

The shell material of *P. purpuratus* reaches the fracture point without plastic deformation. However, when the failure strength of the samples was achieved, the fracture differs depending on the direction of the compression test. Compression across the thickness direction presents a tortuous fracture of the sample, which becomes fragmented into several pieces (pulverized or broken), as shown in Fig. 5a. In contrast, compressions across the longitudinal and transversal directions suggest a pattern in the fracture path, which produced delamination of the sample through a plane $\pi$ (delamination plane), as shown in Fig. 5b. Figure 5c shows a photograph of the sample after the test, which separates into two halves due to the delamination effect. Out of a total of 180 samples of all groups compressed in the directions mentioned above ($\pmb {d}_{l}$ and $\pmb {d}_{tr}$), 95$\%$ of them failed due to delamination at maximum stress. This effect, referred to as “tortuosity” by several authors^4,36,57–61^, has been reported as a toughening mechanism in mollusks proposed by Sarikaya et al.^58^. These studies involve compression tests and directions used in our study, and all showed a similar pattern of fracture types. Their observations were performed using SEM, concluding that the cracks follow the aragonite tiles pattern, known as tortuous fracture path (TFP).

Figure 5a shows the failure of the sample when a load is applied in the thickness direction, a TFP during the crack propagation. Fracture in this direction for nacreous materials has been observed and reported mostly in flexure tests and fracture analysis^1,28,30,62^, where crack propagation advances between the aragonite tiles. Menig et al. reported this type of failure when compression tests were performed on *Haliotis rufescens* shells in the same directions as in our study. The authors observed that failure in the thickness direction suffered crack deflection by organic layers, following the tiles path, while delamination and micro buckling under compression occurred in the other directions^57^. SEM observations were made in two perpendicular planes: the delamination plane $\pi$ of the samples (Fig. 5d), the nacreous layer surface (NS), and on their thickness (Fig. 5e), the nacreous layer cross-section (NC).

Extrapolating this type of failure to other materials, it is possible to find a vast literature on a non-organic brick-and-mortar structure, such as masonry^63–67^. The investigations of these structures include mechanical tests of normal forces to its surface (compression and tension), shear tests, bending, and fracture analysis, in which the failure by compression is applied in the same directions of this study (longitudinal and transversal), and causes a failure similar to the samples of nacreous structures.

**Figure 6:**
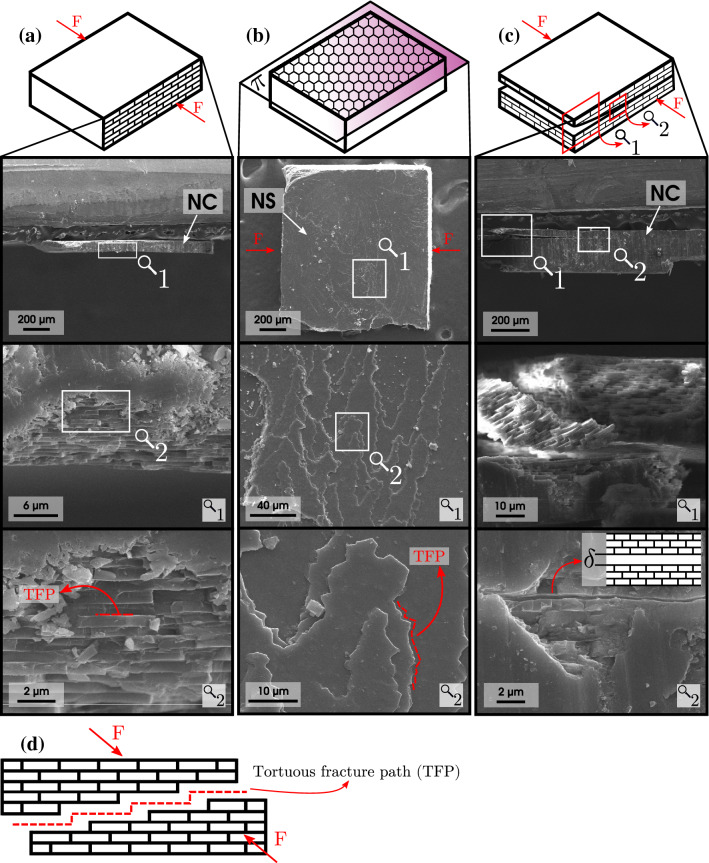
SEM images of the samples at the end of the compression test in the longitudinal ($\pmb {d}_{l}$) and transversal ($\pmb {d}_{tr}$) directions. (**a**) imaging on the layer cross-section (NC), (**b**) Imaging on the nacreous layer surface (NS), (**c**) sample with incomplete delamination, whose fracture origin has an aragonite “bricks” separation of $\delta$, (**d**) tortuous fracture path (TFP). F: direction of the applied force; $\pi$: delamination plane.

Figure 6 shows the SEM images of the samples used in the compression test after fracture in the longitudinal and transversal directions, and their corresponding scales are represented with a magnifying glass symbol. A highly tortuous fracture surface of the nacre is visible in Fig. 6a, extending in the same direction as the applied force (F) on the compressed samples. The microlayers consist of hexagonal tiles, observable through the delamination plane $\pi$ (Fig. 6b). The fracture surface analysis at higher magnification reveals a tortuous fracture path (Fig. 6d), similar to the results reported by Menig et al.^57^. The images reveal that the fracture path’s direction is perpendicular to the force applied in compression (F). This can be corroborated in Fig. 6c, which shows a sample that suffered incomplete delamination in more than one plane in which it is possible to observe the fracture path. At the origin of the crack, there is a separation of the tiles at a distance $\delta$. At the end of the fracture, there is a separation of the aragonite tiles, including a tilted column resulting from the detachment of the material. Other delaminated samples can be seen in Supplementary Fig. S2.

Finally, another aspect to consider of the microstructure of *P. purpuratus* is the thickness of the aragonite tiles. Five tiles of groups 1 and 5 were measured, of which the average was $\sim$0.4 $\upmu$m and $\sim$1.0 $\upmu$m, respectively. This would indicate a possible increase in tile thickness during aging. Liang et al. observed that the *Nautilus* shell possesses a tile thickness of $\sim$330 nm, while the *Cristaria plicata* shell possesses a thickness of $\sim$1.1 $\upmu$m^68^. *Nautilus* lives in a high-pressure environment, and through mechanical testing, they determined that the higher number of layers due to the lower tile thickness in the same volume could be the cause of its shell withstanding such pressures. This could be the possible reason why the macroscopic mechanical properties of *P. purpuratus* would be stronger in the thickness direction in juvenile than in mature individuals.

More detailed measurements are needed in the future to understand interlayer interactions. Considering that the delamination effect was observed in all groups (1–5) and that they suffer an apparent degradation of mechanical properties in the thickness direction (see Fig. 3), it is necessary to analyze in future studies the samples by atomic force microscopy (AFM). This has been done previously on shells of nacreous microstructure to reveal both the structure of a single tablet and its relation to vertically adjacent tablets^69^. This would help relate the material’s microstructure (such as the mineral bridges that hold the aragonite tablets together) to the failure and mechanical degradation of the nacre.

Another interesting factor to consider is the organic matrix. Although the amount of organic composition of P. purpuratus varies from 1 to 5$\%$^2^, studies are needed to evaluate this factor through ontogeny and thus analyze a possible link with the degradation of mechanical properties.

## Conclusions

This research was devoted to characterizing the biomechanical behavior of the *Perumytilus purpuratus* shell by performing quasi-static compression tests. The results indicated a similar performance in the mechanical properties in the longitudinal and transversal directions. However, both are statistically different in the thickness direction, evidencing the orthotropic behavior of the nacreous material. The results also evidenced symmetrical behaviors in elastic modulus, maximum stress, and maximum strain of both valves, and homogeneity in the mechanical behavior across the shell zones. However, the elastic modulus recorded in the three orthotropic directions of the material was negatively affected as the mollusk increases in size/age, although the thickness direction is the one that undergoes more drastic changes; the maximum stress and maximum strain decreasing to a greater extent in the remodeling during the ontogeny. Besides, SEM images of the samples’ microstructure used in the compression tests were obtained to study the material’s failure characteristics. These presented a similar failure mode to the samples reported in studies of nacreous material, a tortuous failure, including delamination in the compressed samples in the longitudinal and transversal directions. Thus, our results indicate that it is relevant to differentiate between the orthotropic directions of the material and the shell size/age of the mollusk used when mechanical properties are evaluated in the thickness direction. In addition, the structural similarity of the shell material to human-made structures (nacreous material and masonry) concerning the failure characteristic is valuable for biomimetics studies. Finally, the deterioration of biomechanical properties in the thickness of older mussels and the failure mode of the shell material when subjected to compression is relevant for understanding prey-predator interactions and environmental forces impacting *P. purpuratus* in valve-exposed habitats. Further studies are necessary to evaluate the stress concentrations required for the shell material delamination and the eco-evolutionary consequences of this performance in mollusk.

## Supplementary Information

Supplementary Information.
