# Supplementary material for: Evaluation of remodeling and geometry on the biomechanical properties of nacreous bivalve shells
Source: Sci Rep. 2022 Jan 13;12:710. doi: 10.1038/s41598-021-04414-1 (PMC8758743; doi:10.1038/s41598-021-04414-1)
Supplement: Supplementary file 1 — Supplementary Information. [file 41598_2021_4414_MOESM1_ESM.pdf]

Supplementary Information
Evaluation of remodeling and geometry on the biomechanical properties of nacreous bivalves shells

Estefano Muñoz-Moya[1], **Claudio M. García-Herrera**[1,*], **Nelson A. Lagos**[2], **Aldo F. Abarca-Ortega**[1,3], **Antonio G. Checa**[4], **and Elizabeth M. Harper**[5]

[1]Departamento de Ingeniería Mecánica, Universidad de Santiago de Chile (USACH), Av. Bernardo O'Higgins 3363, Santiago de Chile, Chile
[2]Centro de Investigación e Innovación para el cambio climático (Ci*i*CC), Universidad Santo Tomás, Av. Ejército Libertador 146, Santiago de Chile, Chile
[3]Center for Biomedical Technology, Universidad Politécnica de Madrid, 28223 Pozuelo de Alarcón, España
[4]Departamento de Estratigrafía y Paleontología, Universidad de Granada, 18071, Granada, España
[5]Department of Earth Sciences, University of Cambridge, Downing Street, CB2 3EQ, Cambridge, UK
[*]claudio.garcia@usach.cl

ABSTRACT

Mollusks have developed a broad diversity of shelled structures to protect against challenges imposed by biological interactions(e.g., predation) and constraints (e.g., pCO_2-induced ocean acidification and wave-forces). Although the study of shell biomechanical properties with nacreous microstructure has provided understanding about the role of shell integrity and functionality on mollusk performance and survival, there are no studies, to our knowledge, that delve into the variability of these properties during the mollusk ontogeny, between both shells of bivalves or across the shell length. In this study, using as a model the intertidal mussel *Perumytilus purpuratus* to obtain, for the first time, the mechanical properties of its shells with nacreous microstructur ; we perform uniaxial compression tests oriented in three orthogonal axes corresponding to the orthotropic directions of the shell material behavior (thickness, longitudinal, and transversal). Thus, we evaluated whether the shell material's stress and strain strength and elastic modulus showed differences in mechanical behavior in mussels of different sizes, between valves, and across the shell length. Our results showed that the biomechanical properties of the material building *P. purpuratus* shells are symmetrical in both valves and homogeneous across the shell length. However, uniaxial compression tests performed across the shell thickness showed that biomechanical performance depends on the shell size (aging); and that mechanical properties such as the elastic modulus, maximum stress, and strain become degraded during ontogeny. SEM observations evidenced that compression induced a tortuous fracture with a delamination effect on the aragonite mineralogical structure of the shell. Findings suggest that *P. purpuratus* may become vulnerable to durophagous predators and wave forces in older stages, with implications in mussel beds ecology and biodiversity of intertidal habitats.

Keywords: Nacre microstructure, Bivalves, Biomechanics, Elastic anisotropy, Mechanical characterization, Fracture

1 Supplementary Information - Statistical

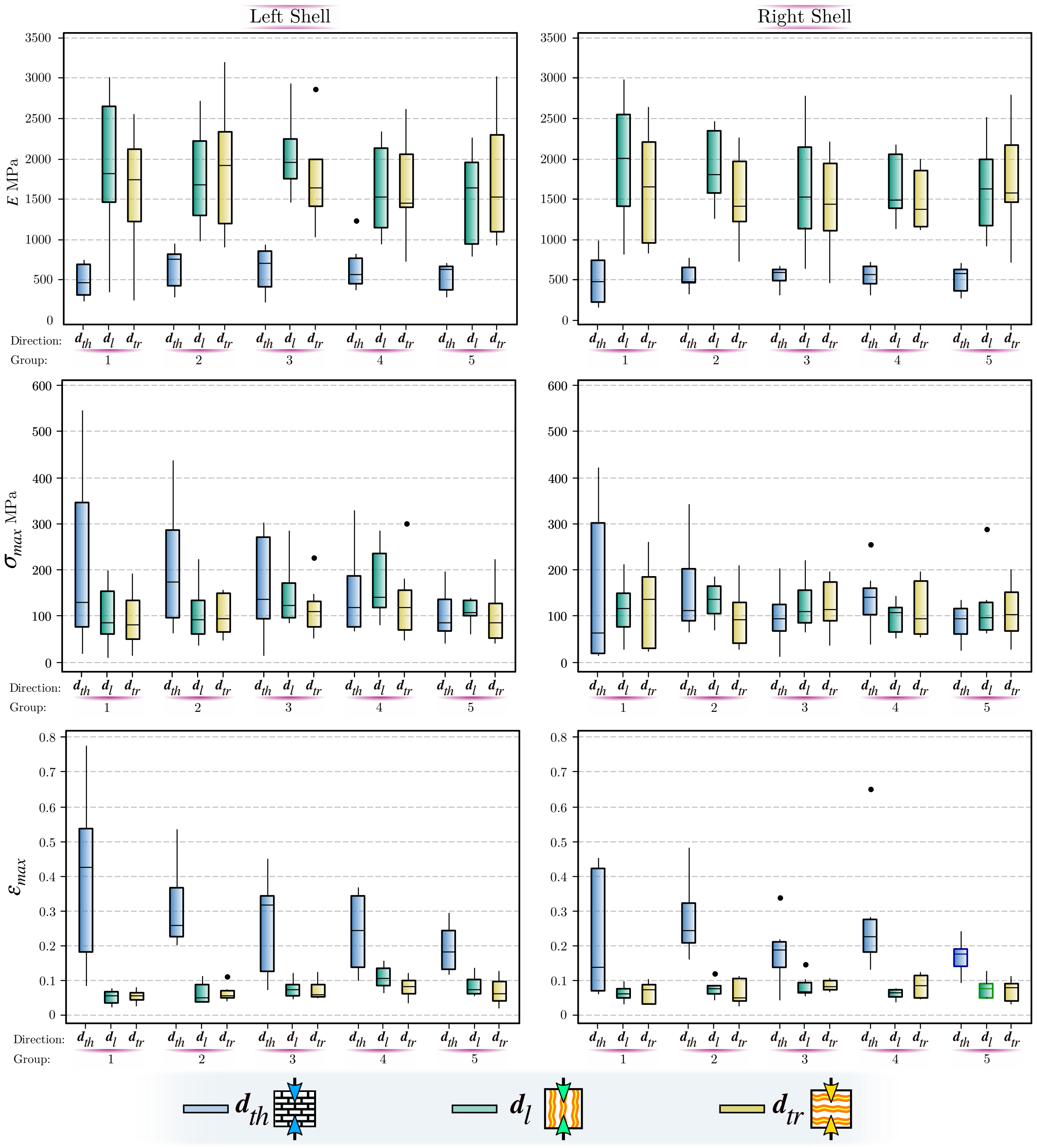

Supplementary Figure S1. Boxplot of elastic modulus (E), maximum stress (σ_{max}), and maximum strain (ε_{max}) evaluated on the left and right shells of *P. purpuratus* for five groups of sizes/ages in the three orthotropy directions: thickness ($\boldsymbol{d}_{th}$), longitudinal ($\boldsymbol{d}_{l}$), and transversal ($\boldsymbol{d}_{tr}$). (●): extreme values of the distribution.

2 Supplementary Information - SEM

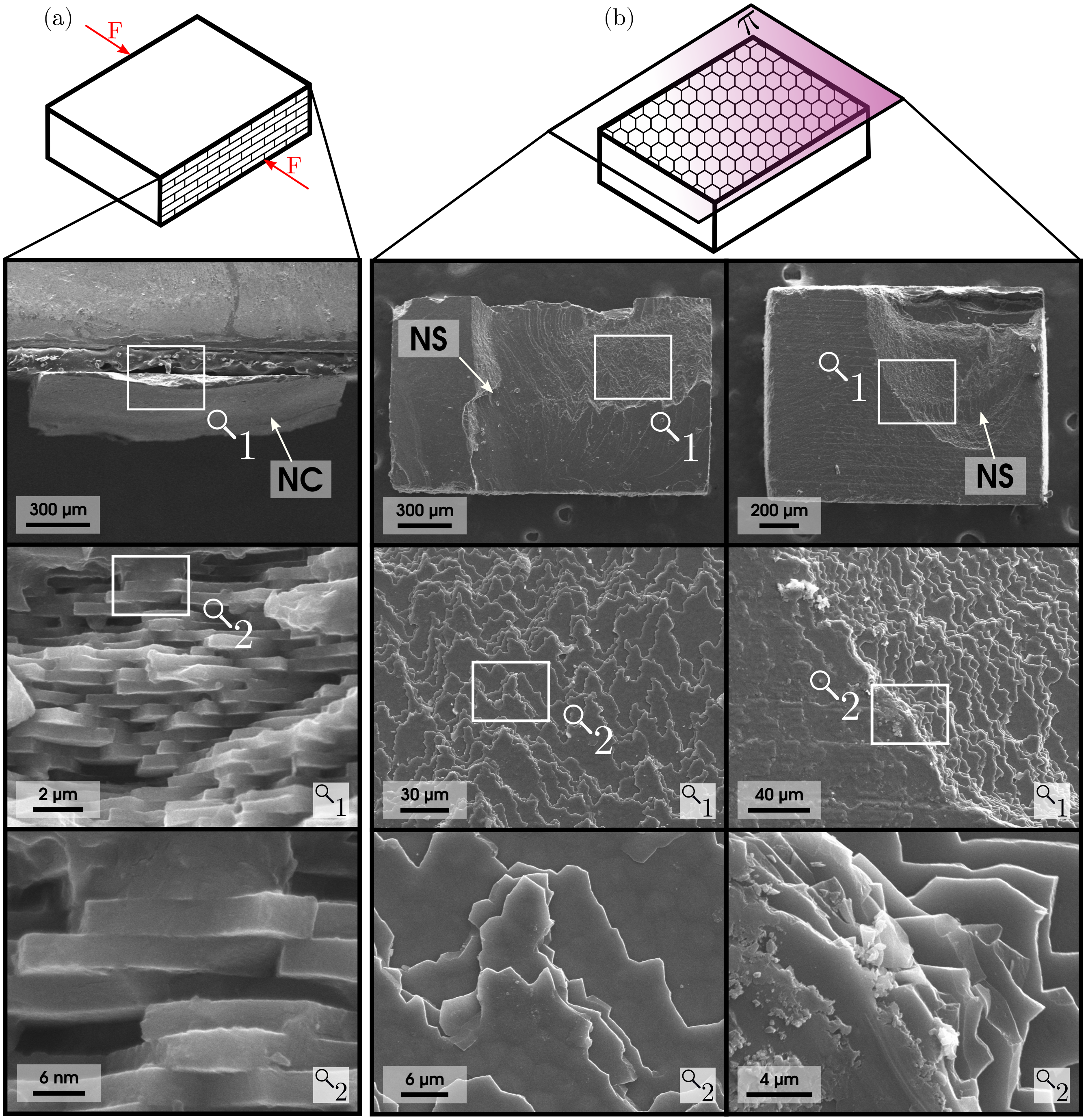

Supplementary Figure S2. SEM images of samples at the end of the compression test in the longitudinal ($\boldsymbol{d}_l$) and transversal ($\boldsymbol{d}_{tr}$) directions. (a) Imaging on the nacreous layer surface (NS), (b) imaging on the layer cross-section (NC). F: direction of the applied force; π: delamination plane.

3 Supplementary Information - Mean value and the standard error

Supplementary Table S1. Mean value and the standard error of the mean (±SEM): elastic modulus (E), maximum stress (σ_{max}) and maximum strain (ε_{max}) for each age/size group and orthotropy direction of the shell material, the thickness ($\boldsymbol{d}_{th}$), longitudinal ($\boldsymbol{d}_{l}$), and transversal ($\boldsymbol{d}_{tr}$) directions.

Group	Direction	E MPa			σ_{max} MPa			ε_{max}		
	$\boldsymbol{d}_{th}$	495.5	±	57.5	178.5	±	38.9	0.307	±	0.049
1	$\boldsymbol{d}_{l}$	1937.5	±	173.2	109.4	±	13.1	0.057	±	0.005
	$\boldsymbol{d}_{tr}$	1643.3	±	154.6	103.8	±	16.7	0.059	±	0.005
	$\boldsymbol{d}_{th}$	591.8	±	50.1	175.9	±	21.7	0.298	±	0.025
2	$\boldsymbol{d}_{l}$	1878.4	±	126.6	122.1	±	11.2	0.069	±	0.006
	$\boldsymbol{d}_{tr}$	1693.5	±	157.8	100.2	±	12.5	0.063	±	0.006
	$\boldsymbol{d}_{th}$	594.8	±	46.2	129.8	±	19.7	0.218	±	0.026
3	$\boldsymbol{d}_{l}$	1817.9	±	139.6	131.3	±	13.2	0.078	±	0.006
	$\boldsymbol{d}_{tr}$	1604.1	±	126.5	117.1	±	11.7	0.076	±	0.005
	$\boldsymbol{d}_{th}$	596.6	±	48.9	137.1	±	16.6	0.252	±	0.030
4	$\boldsymbol{d}_{l}$	1631.9	±	102.3	131.1	±	15.1	0.086	±	0.008
	$\boldsymbol{d}_{tr}$	1545.5	±	108.6	122.0	±	15.2	0.082	±	0.006
	$\boldsymbol{d}_{th}$	517.4	±	36.5	93.8	±	9.8	0.187	±	0.013
5	$\boldsymbol{d}_{l}$	1614.4	±	121.1	114.9	±	12.3	0.078	±	0.006
	$\boldsymbol{d}_{tr}$	1697.2	±	156.7	104.6	±	12.9	0.068	±	0.007
